# Quality of Life Predictors in Patients With Melanoma: A Machine Learning Approach

**DOI:** 10.3389/fonc.2022.843611

**Published:** 2022-03-25

**Authors:** Monica Pinto, Nicola Marotta, Corrado Caracò, Ester Simeone, Antonio Ammendolia, Alessandro de Sire

**Affiliations:** ^1^ Rehabilitation Medicine Unit, Strategic Health Services Department, Istituto Nazionale Tumori-Istituti di Ricovero e Cura a Carattere Scientifico (IRCCS)-Fondazione G. Pascale, Naples, Italy; ^2^ Physical Medicine and Rehabilitation Unit, Department of Medical and Surgical Sciences, University of Catanzaro “Magna Graecia”, Catanzaro, Italy; ^3^ Melanoma and Skin Cancer Surgery Unit, Department of Melanoma, Cancer Immunotherapy and Development Therapeutics, Istituto Nazionale Tumori-Istituti di Ricovero e Cura a Carattere Scientifico (IRCCS)-Fondazione G. Pascale, Naples, Italy; ^4^ Department of Melanoma, Cancer Immunotherapy and Development Therapeutics, Istituto Nazionale Tumori-Istituti di Ricovero e Cura a Carattere Scientifico (IRCCS)-Fondazione G. Pascale, Naples, Italy

**Keywords:** melanoma, quality of life, lymphedema, machine learning, body mass index, cancer rehabilitation

## Abstract

Health related quality of life (HRQoL) is an important recognized health outcome for cancer treatments, but also disease course with slower recovery and increased morbidity. These issues are of implication in melanoma, which maintains a risk of disease progression for many years after diagnosis. This study aimed to explore and weigh factors in the perception of the quality of life and possible relationships with demographic–clinical characteristics in people with melanoma *via* a machine learning approach. In this observational study, patients with melanoma, without metastatic disease, were recruited from January 2020 to December 2021 with a follow-up of at least one year. Demographic variables and clinics were collected, and the 12-Item Short-Form Health Survey (SF-12) was adopted as the physical and mental aspects of the Health-Related Quality of Life (HRQoL) measure. All the variables were processed in a random forest model to weigh at each node of each tree of this machine learning regression model, their actual weight in SF-12 score. We included 203 melanoma patients, mean aged 59.25 ± 15.1 years: 56 (27%) affecting the upper limbs and 147 (73%) affecting the trunk. The model of 142 patients with no missing value, generating 92 trees (MSE = 0.45, R2 of 0.78), reported that the lesion site was the most influencing variable on HRQoL based on the decrease in Gini impurity in variable weighing at each node intersection in forest generation. In this scenario, we built two distinct models for lesion sites and demonstrated that the variable that most influenced the quality of life in upper limb melanoma was lymphedema, while BMI was in the trunk. Given these results, random forest regressions could play a crucial role in the clinical and rehabilitation approach. The machine-learning model for detecting the HRQoL predictor in melanoma patients indicates that the experienced lymphedema and BMI may influence the HRQoL perception. This study suggests that the prevention and treatment of lymphedema and bodyweight reduction might improve the quality of life in melanoma.

## Introduction

Cutaneous melanoma is the most prevalent etiology of skin cancer. In 2021, approximately 101,280 new melanoma cases were predicted in the United States (1, 2). Currently, innovative approaches such as target therapies and immunotherapies have been introduced in clinical practice for the treatment of cutaneous melanoma (3). More in detail, target therapies are based on the use of drugs targeting specific genetic alterations in candidate genes, blocking specific pathways implicated in the oncogenesis of melanoma (3). The most important is the MAP-Kinase (B-RAF/MEK/ERK) pathway (1). Among immunotherapies, checkpoint inhibitors target specific receptors on T-lymphocytes, such as anti-CTL-A4, anti-PD-1/PDL-1, and anti-LAG-3. They all have improved the survival of melanoma patients in both metastatic and adjuvant setting (4, 5). Therefore, we noticed an advancement in the treatment of cancer over the past decade, albeit it could result in an impaired health related quality of life (HRQoL) (6–8).

In this context, beyond the cancer itself, patients with melanoma should undergo follow-up programs and be aware of their condition in order to improve well-being and HRQoL (4, 9). HRQoL has been defined as the ‘perception of the effects of illness and treatment on the physical, psychological, and social aspects of life’ and it is becoming increasingly recognized as an important therapeutic outcome in cancer therapy. Patients seeking medical advice concerning pigmented lesions initially report a good HRQoL, that could often lower by the time of melanoma diagnosis and following treatments (10).

The female sex also predicted greater improvement in HRQoL over time and younger patients (<50 years old) demonstrated poorer HRQoL (11). Moreover, lymphedema is a significant health issue for cancer survivors as a condition that can severely affect the HRQoL of patients (12–14). Sentinel lymph node biopsy (SLNB) has become the procedure of choice for assessing the lymph node status, designed to be less invasive and, hence, less prone to induce complications than a complete lymph node dissection (15, 16). When necessary, lymph node dissections for melanoma treatment is led to rates between 15.7 and 64.3% of secondary lymphedema (5).

Several generic- and specific HRQoL questionnaires have been developed or are under development (6) and are varying in their sophistication, reliability, and validity (17, 18). Validity problems (mainly concurrent and criterion validity) are evident when comparing HRQoL-results between studies, as there is no consensus concerning the definition of the subjective and multidimensional concept of HRQoL (19).

Among traditional statistical methods, logistic regression is the most common when dealing with binary outcomes, like the presence or absence of a variable (20). Conversely, machine learning is a branch of artificial intelligence centered on algorithms which do not need explicit prior programming to function but automatically learn from available data, creating decision models to complete tasks. Random forest regression (RFR) belongs to the branch of the same group of decision trees of machine learning methods, and it might become an essential part of every step of oncological screening strategies and management of patients, thus leading to precision medicine (21).

Therefore, the present study aimed to identify the weight of factors *via* a machine learning approach in the perception of the HRQoL in a cohort of patients affected by melanoma.

## Methods

### Participants

In this observational cross-sectional study, patients referred to the Rehabilitation Medicine Unit, Strategic Health Services Department, Istituto Nazionale Tumori-IRCCS-Fondazione G. Pascale, Napoli, Italy, have been recruited in a 2-year period lasting from January 2020 to December 2021. The inclusion criteria were: confirmed cutaneous melanoma, no metastatic disease, at least 1 year follow-up after melanoma excision and unilateral axillary or inguinal SLNB alone and/or Completion Axillary or Inguino-Femoral Lymph Node Dissection (ALND). The exclusion criteria were: previous ipsilateral Inguino-Femoral surgery, major surgery or previous lymph node surgery to the ipsilateral limb, lymphedema diagnosed before melanoma surgery or during treatment of any cancer or metastatic disease, inpatient hospital treatment within 30 days, heart or kidney failure, or not being mentally fit for inclusion in the study. Data were collected from the medical records of the Rehabilitation Unit Outpatient Clinic in National Cancer Institute of Naples, Italy. All patients completed the SF-12 survey before inclusion in the study. If diagnosed with lymphedema, the stage was I–III according to the International Society of Lymphology (ISL). The diagnosis of lymphedema was based on a detailed clinical history and physical examination (including lymphedema measurements).

The history included onset, development of edema, and symptoms such as swelling, discomfort, feeling of tiredness, and pain in the limb.

The study protocol was approved by the Institutional Review Board and met the guidelines of the responsible governmental agency and the procedures in this study were in accordance with the Declaration of Helsinki, with pertinent national and international regulatory requirements.

### Outcome Measures

Data collected included demographic and clinical information. Demographic variables included age, sex, marital status, and education level. Clinical variables included BMI, lymphedema, SLNB intervention, Axillary or Inguino-Femoral Lymph Node Dissection (ALND), immunotherapy, melanoma location, stage, and number of concurrent comorbid conditions (e.g., diabetes, hypothyroidism, etc.). The 12-item Short-Form Health Survey (SF-12) was used to measure the physical and mental aspects of HRQoL (22, 23). The survey consisted of 12 items measured on five-point scales and has eight subscales: general health, physical functioning, role physical, bodily pain, mental health, vitality, social functioning, and role emotional. Global physical and mental health scores can be calculated using the subscale scores (24).

### Machine Learning

The random forest approach is a machine learning algorithm based on several decision trees, randomly created with “boot-strap samples” from the dataset, to determine the branching of each tree managing predictors at every node point. The forest is based on all influence of the variables at each branch. Therefore, at the same time, all trees influence the estimate for certain weights. Variables with high importance are drivers of the outcome and their values have a significant impact on the forest generation. By contrast, variables with low importance might be omitted from a model, making it simpler and faster to fit and predict (25, 26). There are two measures of importance given for each variable in the random forest. The first measure is based on how much the accuracy decreases when the variable is excluded (25, 26). The second measure is based on the decrease of Gini (IncNodeGini, the purity of the splits of the decision trees) impurity when a variable is chosen to split a node (27). Random forest regression, exploiting lesser variables, omits variables during the calculations to change the explained variance and determines the intrinsic consequence of including or excluding the variables. The final outcome is a rank expressed as mean decrease accuracy (IncMSE%, a measure of sum of squares as a prediction error; the larger the value the larger the importance of a given variable) and mean increase Gini (IncNodeGini, the purity of the splits of the decision trees) (28).

In summary, RF is a machine learning model that estimate the importance of variables based on how best or worse the prediction would be if one or more variables are removed and also it protects the elimination of good predictor variables which are important for the model (29). So, the Gini Variable Importance estimates the importance of individual predictors *via* the changes in the node impurities at each split in each tree in the random forest. This Gini importance or mean decrease in the impurity of the node is the difference between an impurity of a node and the weighted sum of the impurities of the two descendent nodes (30, 31).

In addition to knowing the variable importance, RFR also provides the out-of-bag error rate. Typically, we use about two-thirds of the data from a machine learning sample and the rest is left out. These are known as out-of-bag (OOB) analyses. The estimated error on these omitted samples is known as the OOB error rate. The OOB error rate can be used for validation purposes and for calculating the optimal number of trees required (32). Moreover, the mean square error (MSE) is the mean of the square of the errors. The greater the number, the greater the error. MSE values less than 0.2 express a good fit of the model, values between 0.2 and 0.5 show that the model can predict data relatively accurately, and finally values greater than 0.5 express a model that does not fit. Hence, the lower value of MSE describes the best performance of the model; conversely, the higher value of R^2^ (closer to 1) shows that the regression line fits well with the data and the performance of the model is better.

### Statistical Analysis

Data management and analyses were conducted according to a pre-specified statistical analytical plan. Statistical analysis was performed using the R 3.5.2 software (R foundation, Vienna, Austria). The continuous variables are presented as means ± standard deviations. To ensure homogeneity in the weighing of the variables in the single nodes, we decided to convert the continuous variables into dichotomous ones: i.e., the age of the patients as less than or greater than the median value of 62 years, the body mass index (BMI) of the patients as normal weight (BMI = 18.5–24.9 kg/m^2^) or overweight (BMI >25 kg/m^2^). For each model, the data were split into a training set, which consisted of a random subset representing 80% of the data and a holdout set, comprising the remaining 20%. The random forest package in R was used to perform RFR.

## Results

The final study cohort consisted of 203 patients with melanoma, mean aged 59.25 ± 15.1 years: 56 (27.6%) at the upper limbs and 147 (72.4%) affecting the trunk. A full listing of patient clinical characteristics is listed in **Table 1**.

**Table 1:** Descriptive statistics on the study cohort (n = 203).

Age	*Trunk*	58.81± 15.77
	*UL*	60.41 ± 13.23
	*Overall*	59.25 ± 15.1
Gender	*Male*	67 (33.00)
	*Female*	136 (67.00)
BMI	*Trunk*	28.18 ± 4.42
	*UL*	28.4± 5.20
	*Overall*	28.24 ± 4.62
SLNB	*No*	43 (21.28)
	*Yes*	133 (65.84)
SLNB pN	*Negative*	28 (13.79)
	*Positive*	175 (86.21)
Lymphadenectomy	*No*	4 (1.97)
	*Yes*	195 (96.05)
ALND	*Negative*	113 (55.67)
	*Positive*	90 (44.33)
Immunotherapy	*No*	113 (55.67)
	*Yes*	90 (44.33)
Lymphedema	*No*	68 (33.5)
	*Yes*	135 (66.5)
Stage	*0*	104 (51.23)
	*I*	68 (33.5)
	*II*	29 (14.29)
Hypothyroidism	*No*	189 (93.1)
	*Yes*	14 (6.9)
DM	*No*	178 (87.68)
	*Yes*	25 (12.32)
SF-12 PCS	*Trunk*	37.91 ± 9.3
	*UL*	40.01 ± 8.83
	*Overall*	38.5 ± 9.2
SF-12 MCS	*Trunk*	46.92 ± 10.91
	*UL*	47.83 ± 11.23
	*Overall*	47.17 ± 10.98

ALND, Axillary lymph node dissection; BMI, Body Mass Index; DM, Diabetes Mellitus; SF-12 MCS, Short-form 12 health survey mental component score; SF-12 PCS, Short-form 12 health survey physical component score; SLNB, Sentinel Lymph Node Biopsy; SLNB pN, Sentinel Lymph Node Biopsy Positivity.

The model, built on 142 patients with no missing variable values and relative SF-12 scores, generated 92 trees, contemplating 44 subjects in testing and 36 subjects in validation of machine learning model. So, the MSE value stood at 0.45 with an R2 of 0.78.

The evaluation of the importance of the variables reported that the site of lesion was the most influential variable on the quality of life based on the decrease of Gini impurity when choosing a variable at the junction of a node in the forest generation, demonstrated the importance of the variable as expressed in **Table 2**.

**Table 2:** Variable importance in the entire cohort.

	*Mean decrease in accuracy*	*Total increase in node purity*
Site	0.211	12.525
SLNB	0.113	11.628
Immunotherapy	0.104	9.780
ALND	0.141	9.709
DM	0.129	9.657
BMI	0.102	8.727
Lymphedema	0.100	8.538
Gender	0.088	8.532
Hypothyroidism	0.059	7.330
Age	0.151	7.133
SLNB biopsy positivity	0.063	4.938
Lymphadenectomy	0.009	1.343

The rank is expressed as mean decrease accuracy (a measure of sum of squares as a prediction error); the larger the value the larger the importance of a given variable) and Gini mean increase value (the purity gain of the splits of the decision trees). ALND, Axillary lymph node dissection; BMI, Body Mass Index; DM, Diabetes Mellitus; SLNB, Sentinel Lymph Node.

**Figure 1:**
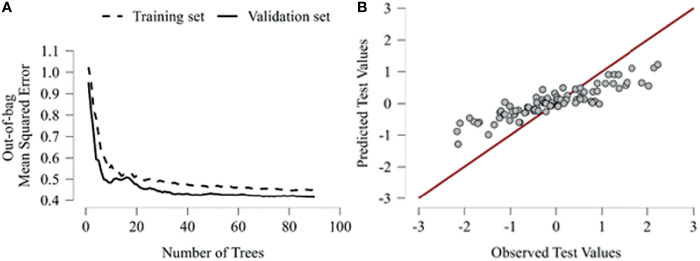
**(A)** Out-of-bag accuracy plots the number of trees against the out-of-bag classification accuracy of the model. The accuracy is evaluated for the training and validation set, as the number of trees generated on the x-axis increases, it is evaluated how the error growths. **(B)** Predictive performance shows the selected test set observations against their predicted values. Thus, the graph analyzes through a hypothetical linear regression how the observed and predicted values correlate.

Given these results, an analysis of the out-of-bag performance of the model assured that as the amount of random trees generation in the forest increased, the curves of the training and validation dataset appear and stand below the MSE less than 0.5, validating the robustness of the prediction. Furthermore, we evaluated the correlation between the predicted values in the model and the actually calculated values. We reported a linear regression index of 0.78, considering a good reliability of the prediction, as depicted in **Figure 1**.

Given the importance of the site variable in the overall analysis, we divided the sample into two subgroups. The UL random forest model, built on 51 patients with no missing variable values and relative SF-12 scores, generated 99 trees, contemplating 12 subjects in testing and 12 subjects in machine learning model validation. Thus, the MSE value stood at 0.49 with an R2 of 0.53.

The importance of the variables assessment regarding UL melanoma showed that lymphedema become the most influential variable on the PCS-SF-12 scores as it increases the precision of the model in purity of the node. On the other hand, the importance of the variable SLNB remains decisive as expressed in **Table 3**.

**Table 3:** Variable importance in patients with upper limb melanoma.

	*Mean decrease in accuracy*	*Total increase in node purity*
Lymphedema	0.244	7.538
SLNB	0.230	5.480
Gender	0.218	5.317
DM	0.092	4.653
Immunotherapy	0.148	4.576
ALND	0.114	4.278
BMI	0.092	2.951
Age	0.116	2.166
Lymphadenectomy	0.040	1.624
SLNB biopsy positivity	0.042	1.458
Hypothyroidism	0.019	0.973

The rank is expressed as mean decrease accuracy (a measure of sum of squares as a prediction error); the larger the value the larger the importance of a given variable) and Gini mean increase value (the purity gain of the splits of the decision trees). ALND, Axillary lymph node dissection; BMI, Body Mass Index; DM, Diabetes Mellitus; SLNB, Sentinel Lymph Node.

Given these results, the out-of-bag performance reported that as the amount of random trees generation in the forest increased, the curves of the training and validation dataset reported an error of 0.87, verifying a minor robustness of the prediction. Furthermore, we evaluated the correlation between the predicted values in the model and the calculated values, reporting a linear regression index of 0.53 with a moderate reliability of the prediction.

Concerning the model on trunk melanoma, built on 91 patients with no missing variable values and relative SF-12 scores, generated 63 trees, contemplating as data split 21 subjects in testing and 16 subjects in validation of machine learning model. The MSE value stood at 0.49 with an R2 of 0.67.

The evaluation of the importance of the variables reported that BMI is the most influential variable on the quality of life as it decreases the precision of the model when the variable is excluded. Similarly, the measure based on the decrease of Gini impurity when choosing a variable at the junction of a node in the forest generation, demonstrated the importance of the variable BMI and SLNB as expressed in **Table 4**.

**Table 4:** Variable importance in patients with trunk melanoma.

	*Mean decrease in accuracy*	*Total increase in node purity*
BMI	0.247	16.531
SLNB	0.153	12.427
Immunotherapy	0.111	8.547
Gender	0.102	7.812
ALND	0.126	7.539
Lymphedema	0.088	6.363
Age	0.145	6.293
DM	0.107	6.028
Hypothyroidism	0.068	5.604
SLNB biopsy positivity	0.057	4.949
Lymphadenectomy	0.001	0.117

The rank is expressed as mean decrease accuracy (a measure of sum of squares as a prediction error); the larger the value the larger the importance of a given variable) and Gini mean increase value (the purity gain of the splits of the decision trees). ALND, Axillary lymph node dissection; BMI, Body Mass Index; DM, Diabetes Mellitus; SLNB, Sentinel Lymph Node.

The analysis of the out-of-bag performance of the learning demonstrated that with increase of random trees generation in the forest, the curves of the training and validation dataset appear and stand with an OBB error of 0.49, verifying the robustness of the forecast. Furthermore, we evaluated the correlation between the predicted values in the model and the calculated values. We reported a linear regression index of 0.67, considering a good reliability of the prediction.

**Figure 2:**
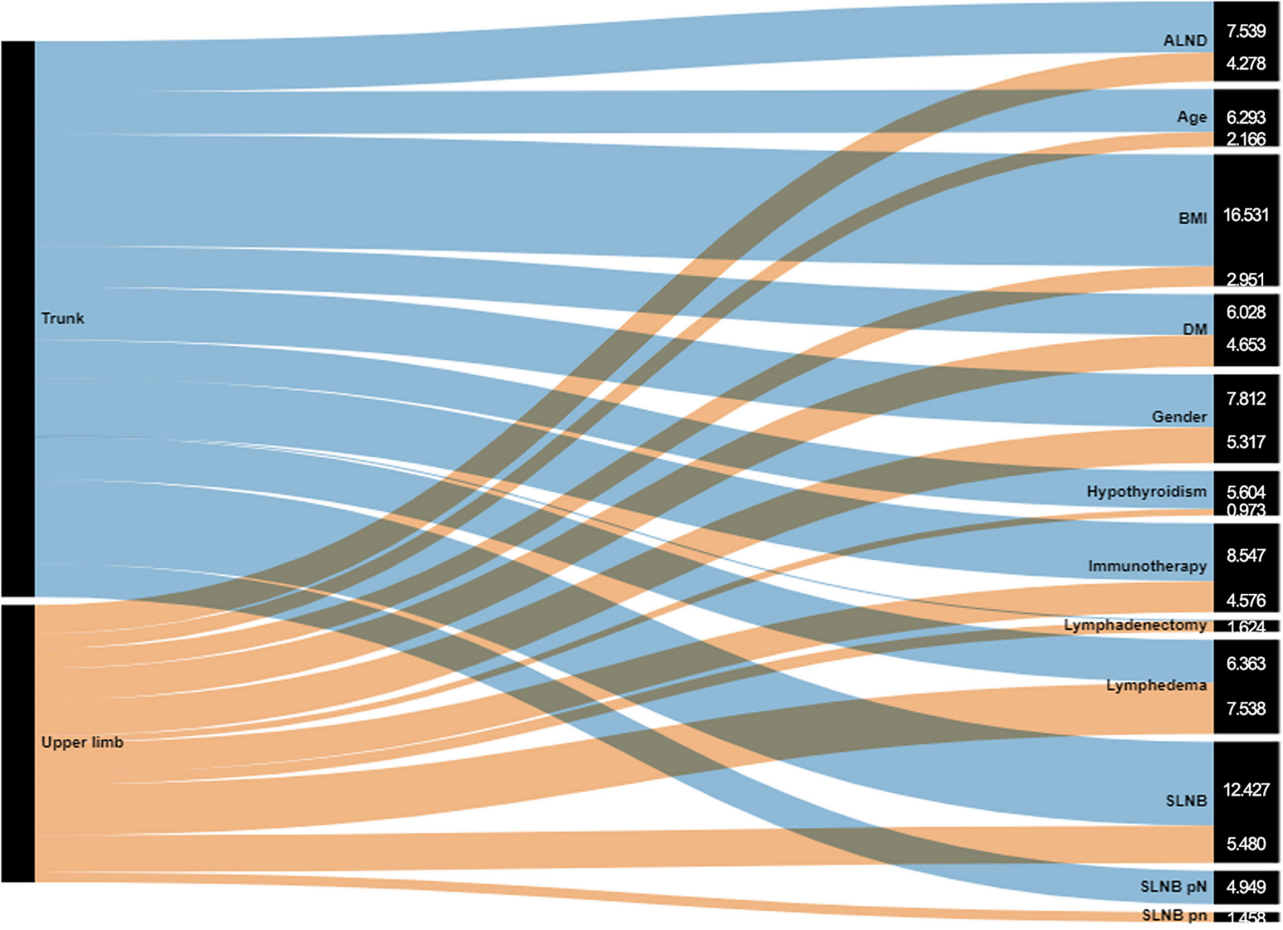
Sankey diagram for total increase in node purity in upper limb and trunk melanoma of variables.

In summary, as shown in **Figure 2**, patients in overall model are affected in HRQoL by the melanoma site and SLNB; on the other hand, the HRQoL of patients with UL melanoma is predominantly influenced by the lymphedema, while the HRQoL of trunk melanoma patients is affected by the weight of the BMI. Intriguingly, positivity of SLNB and age did not seem to influence PCS-SF-12 in a decisive way.

## Discussion

This study aimed to identify the weight of predictors of obtaining PCS-SF-12 in a cohort of people with melanoma through a machine learning approach. The main results reported that the degree of lymphedema and BMI could predict the weight in the perception of HRQoL in patients with upper limb and trunk melanoma, respectively. A melanoma treatment factor such as SLNB influences the quality of life, but not its analysis and positivity, and also age variable which does not substantially affect the machine learning model. Risks of SLNB are low but include a risk for development of lymphedema, although this risk is higher in the lower extremity than in the upper one (33).

Lymphedema is one of the more significant complications that can occur after a lymph node dissection of the axillary or inguinofemoral lymph nodes (34). Usually, these lymph node dissection schemes are performed in the context of node-positive breast cancer and melanoma (35). Secondary lymphedema is a frequent complication after lymphadenectomy in melanoma patients, although few studies in melanoma population adequately characterize risk factors for lymphedema; in addition the sample size is frequently limited (36). Several factors have been implicated in the modulation of lymphatic transport, namely, obesity, age, anatomic location, strength in the contraction of surrounding muscles, damage to sensory nerves, lymphatic leak requiring prolonged drains, hematoma, and wound infections delaying adjuvant treatments (37). Therefore, obesity has demonstrated in preclinical models and in patients to cause decreased lymphatic pumping and leakiness of lymphatics (38, 39).

Indeed, there is a complex relationship between obesity, melanoma, and lymphedema. Preclinical and clinical data showed a correlation between obesity and Breslow thickness, suggesting a higher biologic aggressiveness of melanoma in obese patients (40). Moreover, a recent study has showed that increasing BMI, specifically morbid obesity, is associated with an increased risk of wound complications, namely, lymphedema in the setting of both axillary and inguinal lymphadenectomy (41). In addition, pain at the site of lymph node surgery, and cellulitis of the limb certainly have been reported to increase the risk of lymphedema (42). Given the high number of patients developing lymphedema after inguinal lymph node dissection (ILND), the impact of these chronic issues related to the quality of life and infection on the health care system is not trivial (43, 44). A greater number of lower limb lymphedema symptoms and higher severity may catalyze those negative effects, indicating that proper management of modifiable factors may have a positive impact on patient health and well-being (36). Ryan et al. described the negative experiences of patients with the frustration of symptoms and treatment, diminished self-confidence and poor self-perception, and changes to body image. These findings were further supported by experiences of isolation, depressive thoughts, and fears of the future of lymphedema patients, all of which are implicated in patient HRQoL and psychosocial well-being (45–48). Therefore, Gjourp et al. affirmed that the negative impact of melanoma-related limb lymphoedema on HRQoL was significant in multiple domains of HRQoL (19, 49).

The random forest approach is a machine learning algorithm based on several decision trees, proving its success in both regression and classification problems in recent years and is one of the best machine learning algorithms used in many different fields (50). Developed in the 1990s, random forests have become known for their state-of-the-art capability in classification or regression, and their ability to handle categorical or continuous variables, and in dealing with missing data (51, 52). In addition, in most implementations, the so-called out-of-bag or generalization errors are automatically calculated and their performance is not particularly sensitive to the few hyperparameters that are required to tune the models. Consequently, the popularity of these models in the process industries is growing rapidly, with applications, for example, in predictive modeling.

This study is not free from limitations. Indeed, RFR provides a measure of variable importance, but a current limitation is that no systematic method exists to estimate the shared variances of the variables. Moreover, the mean age of participants included in our cohort was 59.2 years, representing a relatively older cohort of survivors. Although this is representative of the population of people typically diagnosed with cancer, without younger survivors included, the full spectrum of survivorship experiences is not identified. At least, we have included only patients with upper limb and trunk melanoma.

### Conclusions

At the light of our results, the machine learning approach showed that presence of lymphedema and high BMI might influence the perception of HRQoL in patients with melanoma. These findings could contribute to improve interventions supporting symptom management, functioning and improved perception of health status, considering their significant impact on HRQoL.

## Data Availability Statement

The raw data supporting the conclusions of this article will be made available by the authors, without undue reservation.

## Ethics Statement

The studies involving human participants were reviewed and approved by the Istituto Nazionale Tumori Pascale, Naples, Italy. The patients/participants provided their written informed consent to participate in this study.

## Author Contributions

Study conceptualization: MP and AdS. Methodology: NM and AdS. Investigation, MP, CC, and ES. Resources: MP and AA. Formal analysis: NM. Data curation: MP and AdS. Writing—original draft preparation: MP and NM. Writing—review and editing: AdS. Visualization: CC, ES, and AA. Supervision, AA and AdS. Submission: AdS. All authors listed have made a substantial, direct, and intellectual contribution to the work and approved it for publication.

## Conflict of Interest

The authors declare that the research was conducted in the absence of any commercial or financial relationships that could be construed as a potential conflict of interest.

## Publisher’s Note

All claims expressed in this article are solely those of the authors and do not necessarily represent those of their affiliated organizations, or those of the publisher, the editors and the reviewers. Any product that may be evaluated in this article, or claim that may be made by its manufacturer, is not guaranteed or endorsed by the publisher.

## References

[1] Zeng H Liu F Zhou H Zeng C. Individualized Treatment Strategy for Cutaneous Melanoma: Where Are We Now and Where Are We Going? Front Oncol (2021) 11:775100. doi: 10.3389/fonc.2021.775100 34804979 PMC8599821

[2] Garcovich S Colloca G Sollena P Bellieni A Balducci L Cho WC. Skin Cancer Epidemics in the Elderly as an Emerging Issue in Geriatric Oncology. Aging Dis (2017) 8:643–61. doi: 10.14336/AD.2017.0503 PMC5614327 28966807

[3] Crispo A Corradin MT Giulioni E Vecchiato A Del Fiore P Queirolo P. Real Life Clinical Management and Survival in Advanced Cutaneous Melanoma: The Italian Clinical National Melanoma Registry Experience. Front Oncol (2021) 11:672797. doi: 10.3389/fonc.2021.672797 34307142 PMC8298066

[4] Paolino G Cardone M Didona D Moliterni E Losco L Corsetti P. Prognostic Factors in Head and Neck Melanoma According to Facial Aesthetic Units. G Ital Di Dermatol e Venereol (2020) 155:41–5. doi: 10.23736/S0392-0488.17.05685-1 28748684

[5] Patel H Yacoub N Mishra R White A Yuan L Alanazi S. Current Advances in the Treatment of Braf-Mutant Melanoma. Cancers (Basel) (2020) 12(2):482. doi: 10.3390/cancers12020482 PMC7072236 32092958

[6] van Leeuwen M Husson O Alberti P Arraras JI Chinot OL Costantini A. Understanding the Quality of Life (QOL) Issues in Survivors of Cancer: Towards the Development of an EORTC QOL Cancer Survivorship Questionnaire. Health Qual Life Outcomes (2018) 16(1):114. doi: 10.1186/s12955-018-0920-0 29866185 PMC5987570

[7] Invernizzi M de Sire A Lippi L Venetis K Sajjadi E Gimigliano F. Impact of Rehabilitation on Breast Cancer Related Fatigue: A Pilot Study. Front Oncol (2020) 10:556718. doi: 10.3389/fonc.2020.556718 33194622 PMC7609789

[8] de Sire A Losco L Cisari C Gennari A Boldorini R Fusco N. Axillary Web Syndrome in Women After Breast Cancer Surgery Referred to an Oncological Rehabilitation Unit: Which are the Main Risk Factors? A Retrospective Case-Control Study. Eur Rev Med Pharmacol Sci (2020) 24:8028–35. doi: 10.26355/eurrev_202008_22486 32767329

[9] Gaulin C Sebaratnam DF Fernández-Peñas P. Quality of Life in non-Melanoma Skin Cancer. Australas J Dermatol (2015) 56:70–6. doi: 10.1111/ajd.12205 25196191

[10] Al-Shakhli H Harcourt D Kenealy J. Psychological Distress Surrounding Diagnosis of Malignant and Nonmalignant Skin Lesions at a Pigmented Lesion Clinic. J Plast Reconstr Aesthet Surg (2006) 59:479–86. doi: 10.1016/j.bjps.2005.01.010 16749193

[11] Rhee JS Matthews BA Neuburg M Logan BR Burzynski M Nattinger AB. The Skin Cancer Index: Clinical Responsiveness and Predictors of Quality of Life. Laryngoscope (2007) 117:399–405. doi: 10.1097/MLG.0b013e31802e2d88 17334300 PMC1847346

[12] Shaitelman SF Cromwell KD Rasmussen JC Stout NL Armer JM Lasinski BB. Recent Progress in the Treatment and Prevention of Cancer-Related Lymphedema. CA Cancer J Clin (2015) 65:55–81. doi: 10.3322/caac.21253 25410402 PMC4808814

[13] de Sire A Losco L Cigna E Lippi L Gimigliano F Gennari A. Three-Dimensional Laser Scanning as a Reliable and Reproducible Diagnostic Tool in Breast Cancer Related Lymphedema Rehabilitation: A Proof-of-Principle Study. Eur Rev Med Pharmacol Sci (2020) 24(8):4476–85. doi: 10.26355/eurrev_202004_21030 32373985

[14] Invernizzi M Lopez G Michelotti A Venetis K Sajjadi E De Mattos-Arruda L. Integrating Biological Advances Into the Clinical Management of Breast Cancer Related Lymphedema. Front Oncol (2020) 10:422. doi: 10.3389/fonc.2020.00422 32300557 PMC7142240

[15] Wrone DA Tanabe KK Cosimi AB Gadd MA Souba WW Sober AJ. Lymphedema After Sentinel Lymph Node Biopsy for Cutaneous Melanoma: A Report of 5 Cases. Arch Dermatol (2000) 136:511–4. doi: 10.1001/archderm.136.4.511 10768650

[16] Kyrgidis A Tzellos T Mocellin S Apalla Z Lallas A Pilati P. Sentinel Lymph Node Biopsy Followed by Lymph Node Dissection for Localised Primary Cutaneous Melanoma. Cochrane Database Syst Rev (2015) 2015(5):CD010307. doi: 10.1002/14651858.CD010307.pub2 PMC6461196 25978975

[17] Garratt A Schmidt L Mackintosh A Fitzpatrick R. Quality of Life Measurement: Bibliographic Study of Patient Assessed Health Outcome Measures. Br Med J (2002) 324:1417–9. doi: 10.1136/bmj.324.7351.1417 PMC115850 12065262

[18] Krantz E Wide U Trimpou P Bryman I Landin-Wilhelmsen K. Comparison Between Different Instruments for Measuring Health-Related Quality of Life in a Population Sample, the WHO MONICA Project, Gothenburg, Sweden: An Observational, Cross-Sectional Study. BMJ Open (2019) 9(4):e024454. doi: 10.1136/bmjopen-2018-024454 PMC6500231 31005911

[19] Lindqvist Bagge AS Wesslau H Cizek R Holmberg CJ Moncrieff M Katsarelias D. Health-Related Quality of Life Using the FACT-M Questionnaire in Patients With Malignant Melanoma: A Systematic Review. Eur J Surg Oncol (2021) 48(2):312–9. doi: 10.1016/j.ejso.2021.09.013 34600786

[20] Catry FX Rego FC Bação FL Moreira F. Modeling and Mapping Wildfire Ignition Risk in Portugal. Int J Wildl Fire (2009) 18:921–31. doi: 10.1071/WF07123

[21] Cuocolo R Caruso M Perillo T Ugga L Petretta M. Machine Learning in Oncology: A Clinical Appraisal. Cancer Lett (2020) 481:55–62. doi: 10.1016/j.canlet.2020.03.032 32251707

[22] Jakobsson U. Using the 12-Item Short Form Health Survey (SF-12) to Measure Quality of Life Among Older People. Aging Clin Exp Res (2007) 19:457–64. doi: 10.1007/BF03324731 18172367

[23] Younsi M. Health-Related Quality-Of-Life Measures: Evidence From Tunisian Population Using the SF-12 Health Survey. Value Heal Reg Issues (2015) 7:54–66. doi: 10.1016/j.vhri.2015.07.004 29698153

[24] Jenkinson C Layte R Jenkinson D Lawrence K Petersen S Paice C. A Shorter Form Health Survey: Can the Sf-12 Replicate Results From the Sf-36 in Longitudinal Studies? J Public Heal (United Kingdom) (1997) 19:179–86. doi: 10.1093/oxfordjournals.pubmed.a024606 9243433

[25] Sauerbrei W Royston P Binder H. Selection of Important Variables and Determination of Functional Form for Continuous Predictors in Multivariable Model Building. Stat Med 26(30):5512–28. doi: 10.1002/sim.3148 18058845

[26] Sauerbrei W Royston P. Modelling Interactions With Continuous Variables. Das Gesundheitswes (2010) 72:08/09(208). doi: 10.1055/s-0030-1266389

[27] Nembrini S König IR Wright MN. The Revival of the Gini Importance? Bioinformatics (2018) 34:3711–8. doi: 10.1093/bioinformatics/bty373 PMC6198850 29757357

[28] Echeverry-Galvis MA Peterson JK Sulo-Caceres R. The Social Nestwork: Tree Structure Determines Nest Placement in Kenyan Weaverbird Colonies. PloS One (2014) 9(2):e88761. doi: 10.1371/journal.pone.0088761 24551157 PMC3923826

[29] Dharumarajan S Hegde R Singh SK. Spatial Prediction of Major Soil Properties Using Random Forest Techniques - A Case Study in Semi-Arid Tropics of South India. Geoderma Reg (2017) 10:154–62. doi: 10.1016/j.geodrs.2017.07.005

[30] Aldrich C. Process Variable Importance Analysis by Use of Random Forests in a Shapley Regression Framework. Minerals (2020) 10(5):420. doi: 10.3390/min10050420

[31] Louppe G. Understanding Random Forests: From Theory to Practice. arXiv preprint arXiv:1407.7502 (2014).

[32] Leng S Xu Z Ma H. Reconstructing Directional Causal Networks With Random Forest: Causality Meeting Machine Learning. Chaos (2019) 29(9):093130. doi: 10.1063/1.5120778 31575149

[33] Hyngstrom JR Chiang YJ Cromwell KD Ross MI Xing Y Mungovan KS. Prospective Assessment of Lymphedema Incidence and Lymphedema-Associated Symptoms Following Lymph Node Surgery for Melanoma. Melanoma Res (2013) 23:290–7. doi: 10.1097/CMR.0b013e3283632c83 PMC3881422 23752305

[34] Cibula D Borčinová M Marnitz S Jarkovský J Klát J Pilka R. Lower-Limb Lymphedema After Sentinel Lymph Node Biopsy in Cervical Cancer Patients. Cancers (Basel) (2021) 13(10):2360. doi: 10.3390/cancers13102360 34068399 PMC8153612

[35] Herb JN Dunham LN Ollila DW Stitzenberg KB Meyers MO. Use of Completion Lymph Node Dissection for Sentinel Lymph Node-Positive Melanoma. J Am Coll Surg (2020) 230(4):515–24. doi: 10.1016/j.jamcollsurg.2019.12.010 31954818

[36] Bowman C Piedalue KA Baydoun M Carlson LE. The Quality of Life and Psychosocial Implications of Cancer-Related Lower-Extremity Lymphedema: A Systematic Review of the Literature. J Clin Med (2020) 9:1–26. doi: 10.3390/jcm9103200 PMC7601061 33023211

[37] Mailey BA Alrahawan G Brown A Yamamoto M Hassanein AH. Sentinel Lymph Node Biopsy, Lymph Node Dissection, and Lymphedema Management Options in Melanoma. Clin Plast Surg (2021) 48:607–16. doi: 10.1016/j.cps.2021.05.005 34503721

[38] Kataru RP Park HJ Baik JE Li C Shin J Mehrara BJ. Regulation of Lymphatic Function in Obesity. Front Physiol (2020) 11:459. doi: 10.3389/fphys.2020.00459 32499718 PMC7242657

[39] Holder AM Ziemys A. Lymphatic Transport Efficiency Determines Metastatic Potential of Cutaneous Melanoma. Front Oncol (2020) 10:1607. doi: 10.3389/fonc.2020.01607 33042804 PMC7518046

[40] Rutkowski P Indini A De Luca M Merelli B Mariuk-Jarema A Teterycz P. Body Mass Index (BMI) and Outcome of Metastatic Melanoma Patients Receiving Targeted Therapy and Immunotherapy: A Multicenter International Retrospective Study. J Immunother Cancer (2020) 8(2):e001117. doi: 10.1136/jitc-2020-001117 33203662 PMC7674105

[41] Moncrieff MD Sharma RA Gathura E Heaton MJ. Improved Perioperative Seroma and Complication Rates Following the Application of a 2-Layer Negative Pressure Wound Therapy System After Inguinal Lymphadenectomy for Metastatic Cutaneous Melanoma. Ann Surg Oncol (2020) 27:3692–701. doi: 10.1245/s10434-020-08513-7 PMC7471175 32504367

[42] Gjorup CA Dahlstroem K Hendel HW Drzewiecki KT Klausen TW Hölmich LR. Factors Associated With Melanoma-Related Limb Lymphoedema. Acta Oncol (Madr) (2021) 60:779–84. doi: 10.1080/0284186X.2021.1905175 33793386

[43] Jansen MR Vrielink OM Faut M Deckers EA Been LB van Leeuwen BL. One-Year Morbidity Following Videoscopic Inguinal Lymphadenectomy for Stage Iii Melanoma. Cancers (Basel) (2021) 13:1–12. doi: 10.3390/cancers13061450 PMC8004993 33810068

[44] Arié A Yamamoto T. Lymphedema Secondary to Melanoma Treatments: Diagnosis, Evaluation, and Treatments. Glob Heal Med (2020) 2:227–34. doi: 10.35772/ghm.2020.01022 PMC7731060 33330812

[45] Ryan M Stainton MC Jaconelli C Watts S MacKenzie P Mansberg T. The Experience of Lower Limb Lymphedema for Women After Treatment for Gynecologic Cancer. Oncol Nurs Forum (2003) 30:417–23. doi: 10.1188/03.ONF.417-423 12719742

[46] Frid M Strang P Friedrichsen MJ Johansson K. Lower Limb Lymphedema: Experiences and Perceptions of Cancer Patients in the Late Palliative Stage. J Palliat Care (2006) 22:5–11. doi: 10.1177/082585970602200102 16689409

[47] Thomas R Hamilton R. Illustrating the (in)Visible: Understanding the Impact of Loss in Adults Living With Secondary Lymphedema After Cancer. Int J Qual Stud Health Well-being (2014) 9:24354. doi: 10.3402/qhw.v9.24354 25148936 PMC4141939

[48] Winch CJ Sherman KA Smith KM Koelmeyer LA Mackie H Boyages J. “You’re Naked, You’re Vulnerable”: Sexual Well-Being and Body Image of Women With Lower Limb Lymphedema. Body Image (2016) 18:123–34. doi: 10.1016/j.bodyim.2016.06.002 27434105

[49] Rogiers A Leys C Lauwyck J Schembri A Awada G Schwarze JK. Neurocognitive Function, Psychosocial Outcome, and Health-Related Quality of Life of the First-Generation Metastatic Melanoma Survivors Treated With Ipilimumab. J Immunol Res (2020) 28(7):3267–78. doi: 10.1155/2020/2192480 PMC7391091 32775464

[50] Biau G Scornet E. A Random Forest Guided Tour. Test (2016) 25:197–227. doi: 10.1007/s11749-016-0481-7

[51] Schonlau M Zou RY. The Random Forest Algorithm for Statistical Learning. Stata J (2020) 20:3–29. doi: 10.1177/1536867X20909688

[52] Ali J Khan R Ahmad N Maqsood I. Random Forests and Decision Trees. Int J Comput Sci Issues (2012) 9:272–8.

